# DDAM-Net: A Difference-Directed Multi-Scale Attention Mechanism Network for Cultivated Land Change Detection

**DOI:** 10.3390/s24217040

**Published:** 2024-10-31

**Authors:** Junbiao Feng, Haikun Yu, Xiaoping Lu, Xiaoran Lv, Junli Zhou

**Affiliations:** 1 Key Laboratory of Spatio-Temporal Information and Ecological Restoration of Mines of Natural Resources of the People’s Republic of China, Henan Polytechnic University, Jiaozuo 454003, China; fjb2298136535@163.com (J.F.); lxp@hpu.edu.cn (X.L.); lvxiaoran9@163.com (X.L.); 2 Henan Remote Sensing and Mapping Institute, Zhengzhou 450003, China; 15993020695@163.com

**Keywords:** non-agricultural change, high-resolution remote-sensing images, attention mechanism, change detection, self-built dataset

## Abstract

Declining cultivated land poses a serious threat to food security. However, existing Change Detection (CD) methods are insufficient for overcoming intra-class differences in cropland, and the accumulation of irrelevant features and loss of key features leads to poor detection results. To effectively identify changes in agricultural land, we propose a Difference-Directed Multi-scale Attention Mechanism Network (DDAM-Net). Specifically, we use a feature extraction module to effectively extract the cropland’s multi-scale features from dual-temporal images, and we introduce a Difference Enhancement Fusion Module (DEFM) and a Cross-scale Aggregation Module (CAM) to pass and fuse the multi-scale and difference features layer by layer. In addition, we introduce the Attention Refinement Module (ARM) to optimize the edge and detail features of changing objects. In the experiments, we evaluated the applicability of DDAM-Net on the HN-CLCD dataset for cropland CD and non-agricultural identification, with F1 and precision of 79.27% and 80.70%, respectively. In addition, generalization experiments using the publicly accessible PX-CLCD and SET-CLCD datasets revealed F1 and precision values of 95.12% and 95.47%, and 72.40% and 77.59%, respectively. The relevant comparative and ablation experiments suggested that DDAM-Net has greater performance and reliability in detecting cropland changes.

## 1. Introduction

In the 21st century, with the rapid expansion of cities, arable land has continuously changed to non-agricultural usage, posing challenges for the effective use of land resources [1]. Non-agricultural uses include the “misappropriation of arable land” [2], ecological degradation [3], and farmland abandonment [4]. In China, a country with 1.4 billion people, the stability of food production is important to national security [5]. The latest research data show that, in 2020, China’s non-food cultivation area reached 50.71 million hectares, accounting for 30.28% of the country’s arable land area, and this proportion is still rising [6]. This phenomenon indicates an increase in non-agriculturalization behavior, which not only impacts the sustainability of agricultural production but also has a significant impact on national food security [7,8]. To protect the “red line” of 120 million hectares of arable land, the Chinese government and related departments have produced policy guidelines to prevent the encroachment of non-agricultural activities on arable land [9].

The Remote-Sensing Change Detection (RSCD) task entails simultaneously processing dual time-phase images of the same geographic location to identify information about land use/land cover (LULC) changes [10]. Very-High-Resolution Remote (VHR) images have been widely used in the field of natural resources monitoring owing to their superior spatial resolution, short re-entry period, large coverage, and rich texture characteristics [11]. Multi-temporal remote sensing for earth observation enables rapid and precise monitoring of farmland changes, which is critical for farmland conservation, sustainable natural resource management, and social development. High-frequency and large-range monitoring of the non-agricultural behaviors of cultivated land is primarily separated into two categories: (1) CD techniques combining vector data and remote-sensing images; and (2) deep-learning-based CD methods for high-resolution images [12]. In recent years, various academics have confirmed the possibility of merging vector data with remote-sensing images for CD. For instance, Zhao et al. [13] determined the inconsistency of integrating remote-sensing (RS) images with geographic country vectors to demonstrate the viability of segmenting remote-sensing images and extracting more heterogeneous patches, but the required data conditions were more demanding. Liang et al. [14] applied the normalized blue-roof spectral index indirect extraction approach for illegal homes in places with heavy plant cover, but the results exhibited large errors and limited generalizability. In addition, due to the widespread use of machine learning techniques in remote-sensing imaging, certain academics have adopted machine learning methods, including Support Vector Machine (SVM) [15], Decision Tree (DT) [16], Random Forest (RF) [17], and maximum likelihood methods [18]. However, traditional methods rely on artificially manufactured characteristics, which are influenced by threshold and cumulative errors. They also have some limitations in extracting deep information from farmland.

Deep learning methods for RSCD mainly use a fully convolutional network structure, but a fixed-size convolutional kernel produces a limited receptive field, and convolutional networks cannot simulate temporal and spatial contextual aggregation [10]. Recent research has focused on increasing the model’s receptive field by stacking more convolutional layers [19], using inflationary convolution [20], and utilizing the attention mechanism [21]. Peng et al. [22] proposed a method that combines dense skip connections and multilateral output fusion strategies to generate high-spatial-accuracy feature maps by integrating change maps at different semantic levels, which improves the comprehensiveness and change sensitivity of feature extraction. Li et al. [23] propose an end-to-end network architecture that combines the transformer and UNet to achieve higher-resolution multi-scale features and upsample features aggregating through skip connections. At the same time, each pixel is selectively weighted, reducing false alarms and missed alarms. Although these methods achieve feature aggregation through feature concatenation, skip connections, and stepwise upsampling procedures, few research studies consider the multilevel feature interaction that plays a key part in RSCD [24]. To prevent the loss of key features, Lei et al. [25] proposed a difference enhancement (DE) module that effectively reduces the impact of uncorrelated change results by efficiently learning the representation of differences between the foreground and background. Song et al. [26] proposed a Context and Difference Enhancement Network (CDENet) that uses simple cross-layer feature fusion to merge upsampled and high-resolution features, generating change maps by enhancing spatial attention in regions of difference. Zhang et al. [27] used Siamese networks to introduce an attentional mechanism into the deeply supervised parallax discrimination mechanism to fully utilize fine image details and complex texture features in high-resolution images. Zhao et al. [28] built a Triple-Stream Network (TSNet), which uses a dual-stream encoder to extract features from the input bitemporal images and a single-stream encoder to extract features from their concatenated image, yielding deep bitemporal image features and change features and improving the network’s overall CD accuracy. All of the methods mentioned above rely on generating differential features, which inevitably ignores multilevel feature interactions and coupled interactions of key features. Moreover, the image information’s complexity still causes problems with fuzzy detection boundaries and missed target detection.

There are differences between non-agricultural CD and typical remote-sensing monitoring. Deep learning methods for Land Use and Land Cover Change (LUCC) can classify and identify specific types, but they cannot aggregate multiple types into spatial patterns [29]. On one hand, non-agricultural changes on cultivated land in the same area have many different manifestations based on elements such as terrain and environment, light, and natural climate [30]. On the other hand, the progressive improvement in resolution and size of remote-sensing imagery has led to a “scale gap” challenge in the individual features and the entire image [24], especially for non-agricultural programs, where there are built-up regions of varied sizes inside the agricultural area.

The main contributions of this study can be summarized as follows:

(1) To address the problem of insufficient multilevel feature interaction and coupling of key information in the process of CD, we propose a Siamese convolutional network as the backbone to reduce the feature expression of non-changing regions by extracting the difference information in the dual-temporal images through the Difference Enhancement Fusion Module (DEFM). We also introduce the Cross-scalar Aggregation Module (CAM) to aggregate the shallow and deep features of different resolutions through tandem and gradual upsampling. The Attention Refinement Module (ARM) allows the network to pay more attention to the changing regions in the dual-temporal image, which improves the accuracy of non-agricultural CD.
(2) This study proposes an ahierarchical semantic structure for non-agricultural changes in cultivated land, based on historical images of Kaifeng City. A dataset (HN-CLCD) of VHR cropland change was generated to provide trustworthy samples for cropland to buildings, lakes, roads, and greenhouses.

## 2. The Proposed Method

**Figure 1:**
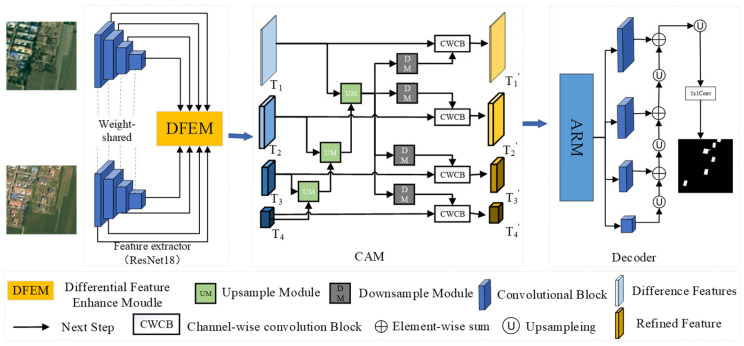
The overall structure of the DDAM-Net network.

This study proposes a new approach, the Difference-Directed Multi-scale Attention Mechanism Network (DDAM-Net), with an encoder–fusion–decoder architecture, which is shown in Figure 1. The encoder’s core is a dual-temporal feature extractor, which uses the Siamese network ResNet18 [31] with shared weights to extract features from the two input images, resulting in a multi-scale representation of distinct levels of features. During the encoding process, feature maps at the same level are interactively processed by the Difference Enhanced Fusion Module (DEFM), generating difference-interaction feature maps rich in semantic information. To efficiently fuse information from different stages and sizes, the network incorporates a Cross-scale Aggregation Module (CAM). This module not only accounts for the multilevel interaction of differential features but also efficiently combines these features, considerably boosting the network’s sensitivity and accuracy for CD. In the decoder step, we use the Attention Refinement Module (ARM) to optimize the fusion of multi-scale and differential features. The ARM concentrates on change regions that have a major impact on detection results using the attention mechanism. Following the refining process, the collected features are stacked and upsampled in a layer-by-layer manner to produce a change map. This allows us to gather change information more properly and improve detection results.

### 2.1. Difference Enhancement Fusion Module (DEFM)

**Figure 2:**
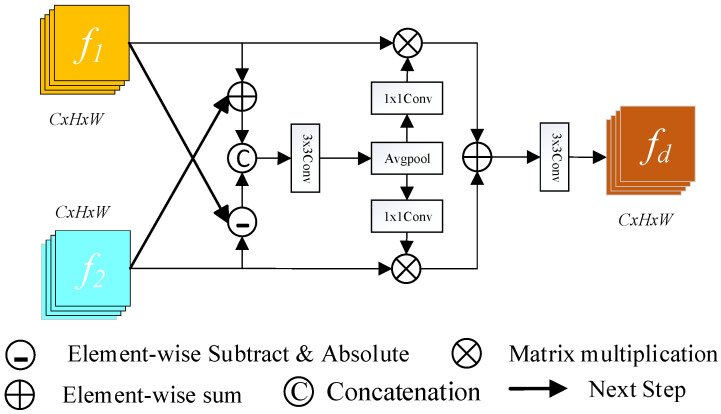
Differential Enhancement Fusion Network (DEFM).

In traditional CD methods, convolutional neural networks extract high-level features such as shape, color, and texture from dual-temporal images using a deep network. However, redundant layers can emerge as the number of network layers rises, resulting in vanishing or expanding gradients [32]. The main idea of the ResNet [31] network is to skip connections, and the application of residual modeling efficiently overcomes the problem of vanishing or exploding gradients, finding a balance between the network depth and model complexity. In this study, we employ the ResNet18 network to extract dual-temporal features and retrieve the structure’s last four levels, which are 4, 8, 16, and 32 iterations of downsampling, as well as 64, 128, 256, and 512 channels of shallow and deep feature maps. To compare features over different periods, this research introduces DEFM, which fully uses the semantic and spatial information retrieved by the main network at each layer using pixel addition and subtraction operations. Generally, CD in remote sensing obtains change information by directly subtracting the pre-image from the post-image; however, this subtraction method frequently leads to serious noise problems, which can interfere with the recognition of changed objects and affect the accuracy of the final results. To address this issue, DEFM is introduced in this study to effectively distinguish between changed and unchanged data and increase the accuracy of subtle difference identification. The architecture of this module is depicted in Figure 2.

First, the DEFM receives two input features, $f_{1}$ and $f_{2}$, taken from the ResNet18 structure. We extract the similar and different features between different tiers of feature maps by performing addition and subtraction operations. Next, a cascade operation is utilized to connect the two features along the channel dimensions for further processing. Then, the generated results are spliced, and a 3 × 3 convolution is performed to obtain the difference weight $f_{w}$. Next, the generated difference weight $f_{w}$ is subjected to average pooling and 1 × 1 convolution, then to a pixel matrix multiplication operation with the original input features. Finally, the obtained findings are sent into a module that undergoes 3 × 3 convolutional processing to extract the difference feature maps of the dual-temporal picture $f_{d}$. This process can be expressed as follows:

(1) $$f_{w}=f_{3\times 3}\left[\left(f_{1}+f_{2}\right)\&\;abs\left(f_{1}-f_{2}\right)\right]$$

(2) $$f_{d}=f_{3\times 3}\left[f_{1}*f_{1\times 1}\left(Avgpool\left(f_{w}\right)\right)+f_{2}*f_{1\times 1}\left(Avgpool\left(f_{w}\right)\right)\right]$$

where $f_{n\times n}$ denotes a convolutional layer with a kernel size of n × n; abs denotes absolute difference operations, which ensures the non-negativity of the change difference features; & denotes tandem operations along the channel dimension; and Avgpool indicates the average pooling.

### 2.2. Cross-Scale Aggregation Module (CAM)

**Figure 3:**
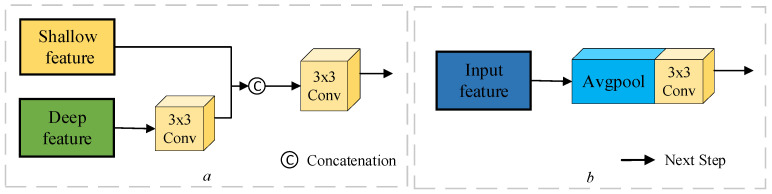
UM is upsampling module (**a**) and DM downsampling module (**b**).

At different stages of feature fusion, the rich semantic information contained in deeper features may influence the accuracy of shallow feature modeling. Chen et al. [33] propose an end-to-end Cross-Scale Feature Fusion (CSFF) framework that can be utilized for feature fusion and augmentation at each feature scale and generate strong and discriminative multilevel feature representations. In this research, characteristics are efficiently aggregated by considering numerous levels of differential feature interactions, as illustrated in Figure 3. The basic function of the upsampling module is to combine the deep and shallow features. The deep features are taken from the early stages of the Siamese network and adjusted to the same size as the shallow features following a 3 × 3 convolution procedure (see Figure 3a). The downsampling module focuses on spatial downscaling, which involves lowering the width and height of the feature map while retaining important feature information. After upsampling the contextual features, the module enhances the overall features of the image via average pooling to avoid relying on local features. It also integrates contextual information of different sizes using 3 × 3 convolution to make the features more representative (see Figure 3b). The CAM suggested in this study processes the multilevel difference feature map $f_{d}$ produced by the DEFM module. After 3 × 3 convolution, the deep feature $T_{i}$ is converted to a convolution kernel of with the same size as the shallow feature. Tandem splicing is then performed with the shallow feature, followed by 3 × 3 convolution again to obtain a feature map with more detailed information. The aggregation characteristics can be expressed as follows:

(3) $$A_{i-1}=Conv_{3\times 3}\left(Cat\left(shallow\;\&\;Conv_{3\times 3}\left(UP\left(deep\right)\right)\right)\right)$$

where shallow denotes shallow features, and deep denotes deep features. After aggregating all features, the final feature, $T_{i-1}$, must be decreased to the same size as $T_{i}$ using average pooling at various scales. The downsampling module smoothes the feature map after average pooling and 3 × 3 convolution to extract higher-level feature information. It then blends aggregated interaction features with difference-enhanced features to generate refined features. In this procedure, Avgpool represents the average pooling operation, whereas CWCB performs channel-level context modeling to accomplish gradual feature abstraction and effective information integration via weighted channels. We apply this strategy to enhance the multilevel disparity features and the refined features derived from the downsampling features, eventually acquiring the refined disparity features ${T_{i}}^{\prime }$.

### 2.3. Attention Refinement Module (ARM)

In the field of CD, typical public remote-sensing image CD datasets frequently suffer from category imbalance [34]. Specifically, in these datasets, the number of unaltered pixels is typically substantially more than that of modified pixels, allowing the model to easily disregard the changed regions during the training phase. To overcome this issue, we offer an attention method that assigns higher weights to modified regions, boosting the model’s detection performance.

In this study, we create ${T^{\prime }}_{\Sigma }$ $\left(i=1\sim 4\right)$ by concatenating four tokens ${T^{\prime }}_{i}$ in series and normalizing the feature dimensions of each layer to avoid the variable-bias effect induced by constant internal updates, where LN represents the layer normalization. Based on the foregoing four tokens, ${T^{\prime }}_{i}$ and ${T^{\prime }}_{\Sigma }$, we compute Query, Key, and Value [35] to obtain the attention weights, which can be expressed as follows:

(4) $$Q={T^{\prime }}_{i}W_{Q},K={T^{\prime }}_{\Sigma }W_{K},V={T^{\prime }}_{\Sigma }W_{V}$$

where $W_{Q}$, $W_{K}$, and $W_{V}$ are the weights of different inputs, and $D_{\Sigma }$ is the channel dimension of the four tokens. A similarity matrix is obtained for weighting using $Q^{T}$ and K. The formula is expressed as follows:

(5) $$MHCA_{i}=Concat\left(\eta_{j}\left[softmax\left(\frac{Q^{T}K}{\sqrt{D_{\Sigma }}}\right)\right]V^{T}\right)/j$$

where Concat represents the concatenation and j represents the number of Multi-head Cross-Attention heads; we use j = 4 in our implementation [36]. The $\eta_{j}$ and softmax functions are employed to normalize the similarity matrix, which helps to smooth the gradient during propagation.

To further enhance the expression of deep features, we focus attention on the channel dimension. In this method, the attention is focused on the channel dimension. For the query Q and the multi-head attention $M_{HCAi}$, we employ the MLP to splice and process them, which can be expressed as follws:

(6) $$Q_{i}=Concat\left(MHCA_{i},MLP\left(Q+M_{HCAi}\right)\right)$$

**Figure 4:**
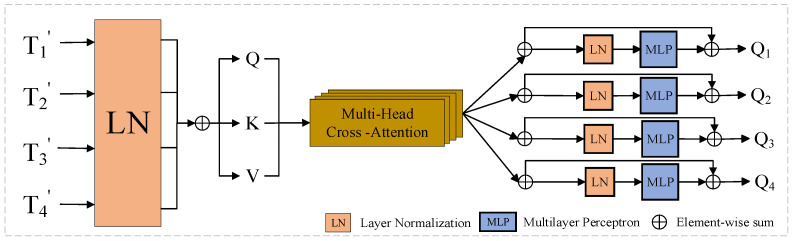
Attention Refinement Module (ARM).

Using this process, we obtained four final outputs: $Q_{1}$, $Q_{2}$, $Q_{3}$, and $\;Q_{4}$, as shown in Figure 4. We used an upsampling technique to stack the difference-enhanced features layer by layer to produce the final variation map. Specifically, the features obtained from difference enhancement processing are upsampling in several layers and gradually fused from shallow to deep layers; eventually, a full change image is created. This approach successfully integrates information across layers, increasing the accuracy and robustness of CD.

### 2.4. Loss Function

The spatial correlation between farmland pixels in RS images refers to the similarity of neighboring pixels in terms of features and information. This property makes the Binary Cross Entropy (BCE) [37] loss function an effective tool for evaluating the model’s performance when analyzing dual-temporal RS images by comparing the predicted and actual results. Furthermore, cropland CD datasets frequently contain much fewer samples in the change category than in the other categories; to solve this issue, balancing adjustments can be performed using the Dice Coefficient (DICE) [38] loss function. In this study, we combine the BCE and DICE loss functions for more accurate model training, which are derived as:

(7) $$L_{loss}=L_{BCE}+L_{DICE}$$

where, $L_{BCE}$ and $L_{DICE}$ are expressed as follows:

(8) $$L_{BCE}=\frac{1}{T}\sum_{k=1}^{T}y_{k}\log\left({\hat{y}}_{k}\right)+\left(1-y_{k}\right)\log\left(1-{\hat{y}}_{k}\right)$$

(9) $$L_{DICE}=1-2\frac{\sum_{k=1}^{H\times W}y_{k}\;\cap \;\sum_{k=1}^{H\times W}{\hat{y}}_{k}\;}{\sum_{k=1}^{H\times W}\;\;\left(y_{k}+{\hat{y}}_{k}\right)}$$

where T represents the total number of pixels in the dual-temporal images, ${\hat{y}}_{k}$ represents the detection result of cropland change in the k image element, $y_{k}$ represents the base case of the k image element, $\sum_{k=1}^{H\times W}{\hat{y}}_{k}\;$ represents the predicted value of cropland change, and $\sum_{k=1}^{H\times W}y_{k}\;$ represents the real value of cropland change.

## 3. Experiment

### 3.1. Dataset

To evaluate the proposed method’s effectiveness in non-agricultural changes, we used three VHR satellite image datasets: PX-CLCD [29], SET-CLCD [39], and the self-built image dataset HN-CLCD. These datasets are from various platforms and sensors, each with a matching binary change mapping label. The HN-CLCD dataset contains several types, mainly used to evaluate the applicability of CD methods in non-agricultural changes. The PX-CLCD and SET-CLCD datasets are two open-source datasets that can serve as important references for testing the generalization ability of current CD methods. Table 1 provides relevant summary information regarding the dataset.

#### 3.1.1. Self-Built Image Dataset HN-CLCD

**Figure 5:**
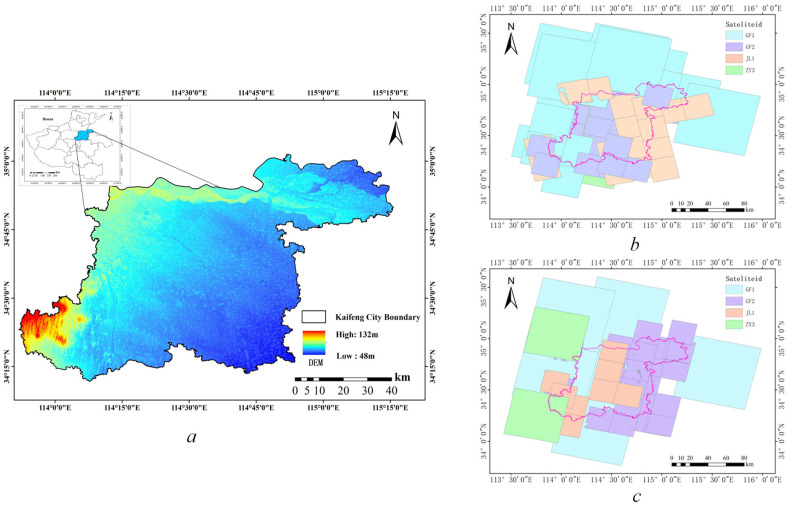
The research area’s geographic location and the different satellite coverage of the before and after images. (**a**) The topographic height of Kaifeng City. (**b**) Image coverage of Kaifeng City in May 2022. (**c**) Image coverage of Kaifeng city in October 2022.

Henan is a typical agricultural province on the upper and middle reaches of the Yellow River, south of the North China Plain. With vast plains and few mountains, it provides one-sixteenth of the country’s cropland and one-tenth of its food [40]. Kaifeng, as a city with large agricultural resources, has a total land area of 6280 square kilometers, with 4340 square kilometers of arable land accounting for 69.20% of the total land area. The area is ideal for growing various crops, such as wheat, corn, cotton, and peanuts, and it is an important food production area. Since the 20th century, Kaifeng City has finished building numerous water-saving projects, such as dams, diversion canals, and land leveling. Large tracts of arid land have been turned into pits or paddy fields due to these actions, and the overall area of arable land has decreased over time with variations [41]. Simultaneously, the demand for construction land has increased, making it the primary driver of non-agricultural uses, and investment in residential and commercial properties is expanding quickly. Food security will therefore be safeguarded by regularly monitoring changes in arable land and balancing the demand for agricultural and non-agricultural land. Figure 5a shows where the research region is located.

In agriculture, there is a relative paucity of intra-class variation datasets. First, the cost of collecting, processing, and standardizing large amounts of information is very high [41]. Second, the changes detected in agricultural land vary significantly due to differences in climate, season, and vegetation type [30]. In this study, we create a non-agricultural changes dataset with local features. The data sources include VHR images from various satellites, classification results of vector data monitored by natural resource management departments, and the land-use classification results of National Land Use Surveying. We chose VHR satellite images, such as JL-1, GF1, GF2, ZY-2, and BJ-2, which cover Kaifeng City, and generated mosaic images for May 2022 and October 2022 after using radiation correction, ortho-rectification, and mosaic functions, as shown in Figure 5b,c. The land cover type of the farmland varies more significantly in these two seasons. When our model detects and achieves high accuracy in this dataset, it indicates a high degree of adaptability for detecting non-agricultural changes in this area or similar environments.

**Figure 6:**
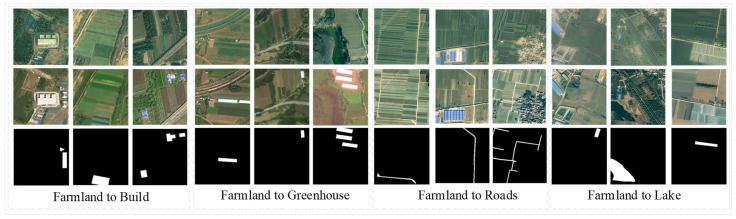
HN-CLCD dataset and its variation types. The area of change is characterized by farmland in the pre-phase and various features such as buildings, sheds, roads, and lakes in the post-phase.

Manually drawing the binary label mappings of cultivable land changes, we created four non-agricultural types: changed cultivable land into houses, changed cultivable land into greenhouses, changed cultivable land into roads, and changed cultivable land into lakes. We obtained 6956 pairs of sample images of 256 × 256 pixels and divided the dataset into training, validation, and test sets in proportions of 80%, 10%, and 10%, respectively. In reality, there are few other samples of changes, and non-agricultural changes are mostly related to buildings in daily life. Overall, the dataset presents an obvious challenge to the problem of class imbalance [42]. Data augmentation is critical to the network’s robustness in tasks with few training tasks. It accomplishes this by increasing the number of small samples through random resizing (between 0.8 and 1.2), random flipping (vertical or horizontal), random cropping, random Gaussian blurring, and random color jitters (brightness = 0.3, contrast = 0.3, saturation = 0.3, hue = 0.3) [43]. Figure 6 shows a partial visualization of the image and ground truth labels.

#### 3.1.2. PX-CLCD Dataset

**Figure 7:**
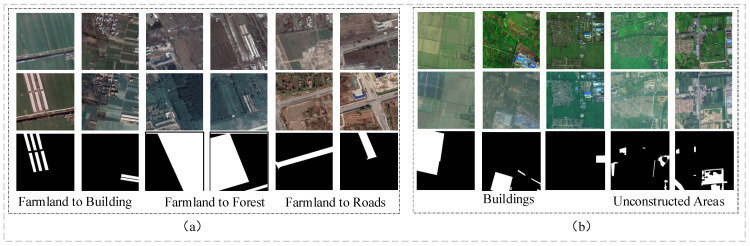
PX-CLCD dataset (**a**) and SET-CLCD dataset (**b**).

The PX-CLCD [29] dataset consists of 5170 pairs of RGB images and the corresponding binary-altered label mappings, as shown in Figure 7a. The research area is located in Peixian, Jiangsu Province, China, and was captured by the Gaofen-2 satellite in 2018 and 2021. The satellite image sources for PX-CLCD and the self-built dataset HN-CLCD are completely different. This can verify the applicability of the non-agricultural model proposed in this study to different star sources. Land changes include new buildings, new forest land, and new highways on cultivated land.

#### 3.1.3. SET-CLCD Dataset

The SET-CLCD dataset [39] is a competition dataset used for the 2022 International Remote Sensing Image Intelligent Processing Algorithm Competition (2022 RSIIPAC). As shown in Figure 7b, the dataset focuses on change targets of all sizes, including the addition and demolition of buildings.

### 3.2. Evaluation Metrics

This study evaluates the efficacy of the model on the dataset using four evaluation indices: precision (PR), recall (RC), F1-score (F1), and intersection over union (IoU). The formulations are as follows:

(10) $$precision=\frac{T_{p}}{T_{p}+F_{p}}$$

(11) $$recall=\frac{T_{p}}{T_{p}+T_{n}}$$

(12) $$F1=\frac{2\cdot precision\cdot recall}{precision+recall}$$

(13) $$IoU=\frac{T_{p}}{T_{p}+F_{p}+F_{n}}$$

where $T_{p}$ indicates the number of pixels successfully identified as changing areas, reflecting the model’s ability to capture changes in the farmland; $F_{p}$ indicates the number of misdiagnosed pixels, illustrating the fact that regions that truly remain unchanged are misclassified as changing areas; $T_{n}$ indicates the number of pixels correctly identified as unchanging areas, reflecting the demonstration of the model’s effectiveness in identifying stable areas; and $F_{n}$ indicates under-reporting, incorrectly identifying a truly changing area as an unchanged region.

### 3.3. Experiment Setting

To train the network, all of the experiments in this study used the PyTorch framework and an NVIDIA GeForce RTX 2080S GPU. Gradient descent was performed using the Adam optimizer, with a weight decay of 0.001 and momentum of 0.9. During training, the batch size was set at eight, and the learning rate was set to 0.001.

## 4. Experiment and Results

### 4.1. Comparison of Most Recent Networks

To validate the effectiveness and superiority of DDAM-Net, we used several previous CD methods to compare the three datasets (PX-CLCD, SET-CLCD, and HN-CLCD). The selected networks are characterized as follows:

FC-EF [44] uses the UNet architecture to obtain a tighter comparison by linking the dual time-phase images in series as inputs. This allows the model to treat the two images as separate channels. SNUNet [45] is based on the U-Net++ architecture and incorporates the Channel Attention Module (ECAM) to extract and enhance features from multiple levels. This approach excels at CD in complex scenes and is especially suitable for dealing with changes in high-complexity backgrounds. STANet [21] employs ResNet-18 as the backbone network and introduces a spatiotemporal self-attention mechanism to deal with complex circumstances and establish global contextual information, highlighting changing regions more precisely. LightCDNet [46] is a lightweight network model that uses DSFM as its basic component. It aims to reduce the number of parameters while retaining performance equivalent to current mainstream technologies. HCGMNet [47] extracts coarse characteristics of the dual-temporal image using the normalized VGG-16 network as the backbone, then adopts a CGM, a self-attention method to gain a broader sensory field, and eventually refines the edge features through hierarchical modifications. CGNet-CD [48] successfully led the fusion of multi-scale features by integrating the Context Guided (CG) self-attention module as a priori information, improving the model’s ability to detect small changes.

### 4.2. Experiments on Self-Built Dataset

Table 2 displays the results of the quantification of HN-CLCD using various methods. FC-EF has the lowest performance on the dataset, with F1 and IoU scores of only 64.73% and 47.85%, respectively, yet the indicators of numerous other methods improved. SNUNet and STANet are made up entirely of Siamese Net and attention modules, with no skip connections between the encoder and decoder to fuse information at various scales, resulting in low model quantization accuracy. Compared to them, LightCD can improve fused feature guidance by efficiently integrating various scale features in the encoder, with F1 values increasing by 2.43% and 3.27%, respectively. Compared to HCGMNet and CGNet-CD, the values of F1 and IoU of DDAM-Net are improved by 5.07%, 6.67%, and 3.13%, 4.18%, respectively, which is attributed to the fact that our method uses DEFM in the encoder to extract the difference information of the dual-temporal images. This not only reduces pseudo-variations caused by the superposition of information channels but also introduces the ARM, which adequately mines the relationship between contextual difference features and generates more detailed feature information from deep to shallow.

**Figure 8:**
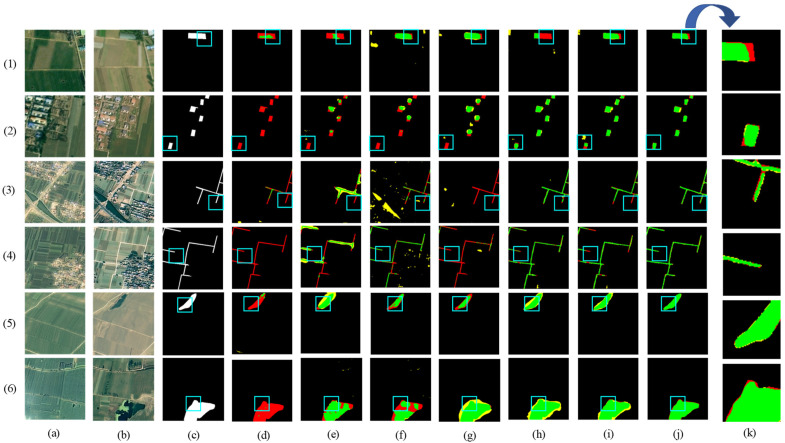
Comparison of change maps of different methods on the HN-CLCD dataset. (1) Greenhouse. (2) Buildings. (3–4) Roads. (5–6) Lakes. (**a**,**b**) Input bitemporal images. (**c**) Label. (**d**–**j**) Change maps of FC-EF, SNUNet, STANet, LightCD, HCGMNet, CGNet-CD, DDAM-Net. (**k**) represents a localized zoomed-in view of the box. Green means correct detection, denoted by $T_{p}$. Red means missing detection, denoted by $F_{n}$. Yellow means false detection, denoted by $F_{p}$.

Figure 8 shows the visual results of the various methods used for predicting the HN-CLCD dataset. All benchmark networks exhibit incompleteness and fragmentation issues in change region detection. SNUNet and STANet perform well in identifying non-agricultural types, such as greenhouses or lakes, but suffer from varying degrees of omissions and misdetections in predicting small buildings and roads (e.g., Figure 8e,f), indicating that the dataset has an imbalance of positive and negative samples. LightCD predictions demonstrate strong aggregation for buildings and lakes but significant difficulties in predicting narrow feature classes (e.g., (3), (4) in Figure 8). HCGMNet, CGNet-CD, and DDAM-Net can all identify fine-scale changes in the cultivation area (e.g., (2), (4) in Figure 8), but there is a large bias in the prediction of lake boundaries when using HCGMNet and CGNet-CD (e.g., (5), (6) in Figure 8). In contrast, our model DDAM-Net can better predict the boundaries of a relatively small number of roads and lakes in the dataset (e.g., (3)–(6) in Figure 8).

### 4.3. Generalization Experiments on PX-CLCD Dataset

Table 3 displays the quantitative results of various methods used on the PX-CLCD dataset. FC-EF has the lowest F1 and IoU of the researched methods, 64.03% and 47.09%, respectively. Unlike the STANet model containing the self-attention mechanism, SNUNet uses rationing between different weights of channel attention to improve F1 and IoU by 10.65% and 12.01%, respectively, indicating the importance of channel attention in global feature extraction. LightCD adds time attention and multi-feature fusion to lightweight networks, significantly improving the CD feature extraction capability of early fusion approaches. HCGMNet, CGNet-CD, and DDAM-Net make great progress in various metrics by using a multi-scale fusion method between the encoder and decoder in the CD branch. Compared to HCGMNet and CGNet-CD, our model DDAM-Net has the best performance in terms of accuracy quantification, with F1 and IoU improvements of 4.09%, 7.06%, and 0.86%, 1.56%, respectively. This is due to HCGMNet and CGNet-CD using multi-scale feature fusion, while our network DDAM-Net uses a multi-scale differential fusion strategy.

**Figure 9:**
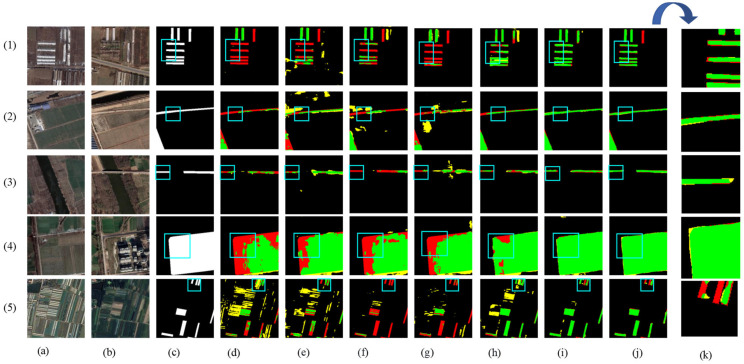
Comparison of change maps of different methods on the PX-CLCD dataset. (1) Buildings. (2–3) Roads. (4–5) Forest. (**a**,**b**) Input bitemporal images. (**c**) Label. (**d**–**j**) Change maps of FC-EF, SNUNet, STANet, LightCD, HCGMNet, CGNet-CD, DDAM-Net. (**k**) represents a localized zoomed-in view of the box. Green means correct detection, denoted by $T_{p}$. Red means missing detection, denoted by $F_{n}$. Yellow means false detection, denoted by $F_{p}$.

The results of the partial visualization of the model in the PX-CLCD dataset are shown in Figure 9, where the FC-EF, SNUNet, STANet, and LightCD networks have many false and missed alarms when predicting changing forest and roads in high-density farmland. The ability to detect partial classes is unsatisfactory, especially in forests (e.g., (2), (3), (5) in Figure 9). Compared to HCGMNe, LightCD has great advantages in upgrading large structures but is less effective in predicting a larger number of tiny buildings in farming (e.g., (1) in Figure 9). HCGMNet, CGNet-CD, and DDAM-Net can perceive the richness of low- and high-level features, and their prediction accuracies for bigger intra-class differences in farms are, in general, better than the other networks (e.g., Figure 9h–j). Among them, our network DDAM-Net is accurate in the prediction of roads and major buildings, and the prediction result map is most similar to the image labels despite the missed-alarms problem (e.g., (1), (5) in Figure 9), demonstrating the effectiveness and robustness of our proposed model.

### 4.4. Generalization Experiments on SET-CLCD Dataset

Table 4 shows the quantitative results from the SET-CLCD dataset. Due to the presence of targets with imbalanced sizes and samples with insignificant changes in the ground truth labeling of the dataset, the model was unable to achieve a high score of detection results on the dataset. The benchmarking network FC-EF has the lowest F1 and IoU at 58.30% and 41.14%, respectively, indicating a need for greater detail and advanced algorithms. In addition, LightCD, HCGMNet, and CGNet-CD achieve significantly higher accuracy than the other benchmark networks, all of which use the attention mechanism to strengthen the connection between multi-scale fusion features. HCGMNet has the highest recall of the benchmark models, at 67.89%, indicating that it predicts positive samples more correctly than the other networks. DDAM-Net outperformed other models on the SET-CLCD dataset, with accuracy, F1, and IoU scores of 77.59%, 72.40%, and 56.74%, respectively.

**Figure 10:**
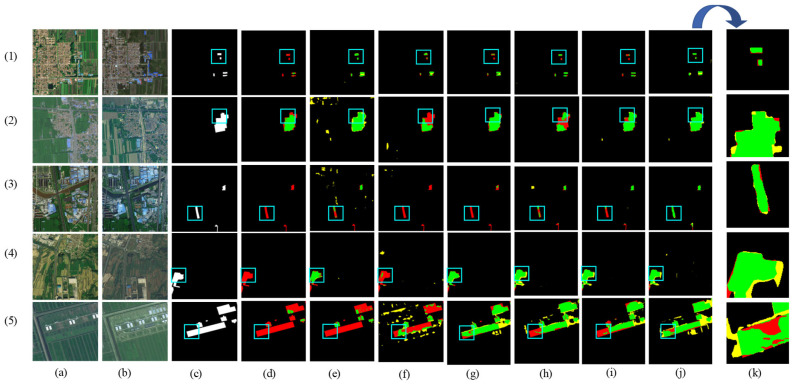
Comparison of change maps of different methods on SET-CLCD dataset. (1)–(3) Large- and small-buildings areas. (4), (5) Unconstructed areas. (**a**,**b**) Input bitemporal images. (**c**) Label. (**d**–**j**) Change maps of FC-EF, SNUNet, STANet, LightCD, HCGMNet, CGNet-CD, DDAM-Net. (**k**) represents a localized zoomed-in view of the box. Green means correct detection, denoted by $T_{p}$. Red means missing detection, denoted by $F_{n}$. Yellow means false detection, denoted by $F_{p}$.

Figure 10 shows the results of the model’s partial visualization in the SET-CLCD dataset, where some of the baseline networks, including FC-EF, SNUNet, and STANet, have serious target-missed alarms (e.g., (3) in Figure 10). These methods cannot overcome the problem of pseudo-change caused by complex buildings on cultivated land. Compared with LightCD and DDAM-Net, HCGMNet and CGNet-CD have relatively poor applicability between buildings and unconstructed areas in farmland (e.g., (2) and (5) in Figure 10) but have high accuracy in predicting unconstructed areas on croplands with large differences in light and dark (e.g., (4) in Figure 10). Furthermore, DDAM-Net can demonstrate the network’s superiority through multi-feature fusion and differential enhancement, which retains excellent prediction ability in small-buildings areas and unconstructed areas, as well as increasing fine-grained spatial segmentation (e.g., Figure 10j). However, it suffers from higher false detection in the prediction of large buildings compared to the LightCD network (e.g., (5) in Figure 10).

### 4.5. Model Efficiency Analysis

**Figure 11:**
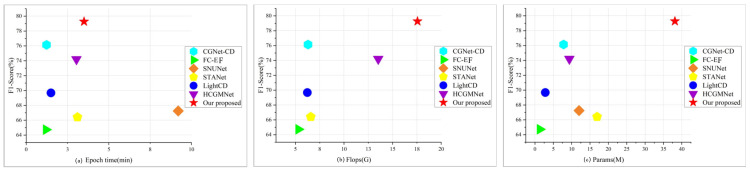
Quantitative analysis of the performance of different models: (**a**) shows F1 of the model versus the time required to run the epoch; (**b**) shows the F1 of the model and the amount of computation; (**c**) shows the number of parameters and the F1 of the model.

The goal of this study is to achieve high-precision non-agricultural detection in cultivated land, and we conducted a thorough quantitative analysis of our proposed DDAM-Net model by comparing metrics such as Params, Flops, and the time required to perform each epoch on the dataset, as shown in Figure 11. Overall, FC-EF has advantages in terms of runtime speed and parameter count, but its small number of feature layers results in low accuracy. Models incorporating attention methods, such as SNUNet and STANet, improve accuracy to some extent but dramatically increase the runtime. Lightweight and efficient network models, such as LightCD, HCGMNet, and CGNet-CD, maintain high accuracy while lowering the number of parameters. However, while DDAM-Net performs comparably to other baseline models in terms of F1 and training efficiency, its parameter count remains high across all models, indicating that more research into lightweight frameworks is needed.

**Figure 12:**
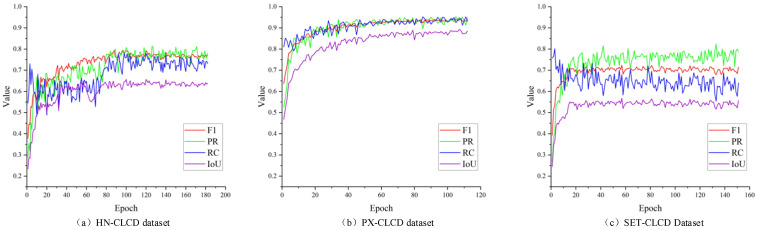
The variation of DDAM-Net’s PR, RC, F1, and IOU metrics on different validation datasets with increasing number of trainings, (**a**) on the HN-CLCD dataset, (**b**) on the PX-CLCD dataset, and (**c**) on the SET-CLCD dataset.

In this study, we trained the model for over 100 epochs, and Figure 12 depicts the accuracy metrics of DDAM-Net on multiple validation datasets. The results reveal that the model performs the best on the PX-CLCD dataset, with all metrics (PR, RC, F1, and IoU) reaching high levels. This excellent performance reflects the good labeling quality and high prediction accuracy of the PX-CLCD dataset. Comparatively speaking, the performance on the SET-CLCD dataset is inferior, which may be related to the sample size, sample diversity, and labeling quality of this dataset. Nonetheless, it is worth noting that our dataset outperforms other datasets in terms of picture star source selection, since it covers a broader range of scenes and target classes. This diversity provides valuable training data for model learning and improves its adaptability to various situations and targets. However, the current dataset’s very low recall rate indicates that the model cannot distinguish positive samples. Future work could focus on improving the recall rate for in-depth exploration, which could include increasing sample diversity, improving the model architecture, and implementing more advanced training strategies, all with the goal of improving the model’s overall performance and practical application ability.

## 5. Ablation Experiment

The DDAM-Net model consists of DEFM, CAM, and ARM modules. To assess the significance of the three modules proposed in this research, we conducted an ablation experiment on the HN-CLCD dataset. The experimentation involved gradually adding each module to the backbone network and then conducting qualitative and quantitative analysis. The experimental results are shown in Table 5.

(1) Ablation experiments for Model-a: This method is the base module used for comparison, and it is made up of the Resnet backbone network and the DEFM module.
(2) Ablation Experiment for Model-b: Compared to the Model-a design, this design aims to evaluate whether the ARM can direct the network to pay more attention to the regions that have changed, hence preferentially assigning weights to the changed areas. The experimental results suggest that adding the ARM increases the network’s F1 and IoU accuracies on the HN-CLCD dataset by 4.35% and 5.49%, respectively.
(3) Ablation Experiment for Model-c: To evaluate the significance of different stages of feature maps in the CD, we created an ablation experiment for the CAM. The experimental results show that the introduction of CAM improves the model’s multi-stage interactions for features of different sizes when compared to the Model-a design, resulting in an increase in the F1 and IoU precision of the network by 2.89% and 3.61%, respectively. The model has the highest recall of 76.32%, implying that adding the CAM module helps reduce missed detections in CD.
(4) Ablation Experiment for Model-d: To verify whether DEFM enables the model to effectively divide the changing and unchanging regions, we designed an ablation experiment for Model-d. Compared with the Model-e design, the added DEFM module improves fusion capabilities for different features, and the F1 and IoU accuracies of the network are improved by 5.81% and 7.6%, respectively.

**Figure 13:**
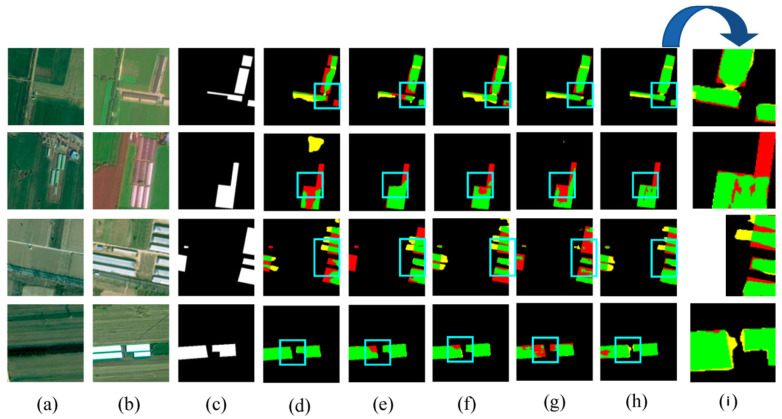
Visualization of ablation study on HN-CLCD dataset. (**a**) Image1. (**b**) Image2. (**c**) Label. (**d**) Model-a. (**e**) Model-b. (**f**) Model-c. (**g**) Model-d. (**h**) Model-e. (**i**) represents a localized zoomed-in view of the box. Green means correct detection, denoted by $T_{p}$. Red means missing detection, denoted by $F_{n}$. Yellow means false detection, denoted by $F_{p}$.

Figure 13 provides visualized comparisons of the ablation results. As shown in the example results, while there are some pseudo changes in Model-b’s visualization results, they are closest to the predictions of the DDAM-Net model, demonstrating the importance of the ARM module in guiding the model to focus on similar and different features. Although Model-c has more false detections, it has fewer missed detections than Models-b and Model-d, demonstrating that the CAM’s multilevel feature interactions are beneficial in reducing CD misses, which is critical for non-agricultural CD approaches. In conclusion, the DEFM, CAM, and ARM proposed in this paper can effectively improve the accuracy of the CD model, and the integration of the three modules obtains the best results in the ablation experiments.

## 6. Conclusions

In this study, we collected multi-measurement data from Kaifeng City, Henan Province, and used geographical and national monitoring classification criteria to define farmland change types, resulting in a dataset of non-agricultural changes, HN-CLCD. Then, we proposed DDAM-Net, a CD network based on differential fusion that uses a Siamese architecture to extract multilevel features from dual-temporal images and DEFM to perform pixel addition and subtraction operations on semantic and spatial information at the same level, reducing redundant information caused by feature fusion and efficiently distinguishing between similar and dissimilar features. Second, the CAM receives multiple levels of different features, and the multilevel feature interaction between deep and shallow features improves the detection of changing regions. The ARM dynamically adjusts the weights between channels and pixels, allowing the network to focus on changing regions in the dual-temporal phase image and improve edge detail features.

Compared with other methods, our proposed method tends to improve the accuracy of the classification of non-agriculturalization projects. On the self-built dataset HN-CLCD, the F1 of DDAM-Net is 79.27%, which is 5.07% and 3.13% higher than the F1 accuracy of HCGMNet and CGNet-CD. In generalization examinations, the DDAM-Net model’s F1 on the publicly accessible datasets PX-CLCD and SET-CLCD was 95.12% and 72.40%, respectively, with 95.47% and 77.59% precision. This study’s results further illustrate the effectiveness and superiority of DDAM-Net in non-agricultural change programs.

As a continuation of this work, the shortcomings of this study are concentrated in the following areas:

1. In this study, the monitoring of non-agriculturalization of farmland is mostly based on deep learning algorithms that extract features from optical images. However, these algorithms are not always effective at differentiating between identical feature types [49]. In contrast, hyperspectral or multispectral imaging techniques can considerably reduce the effect of illumination variations on target features, enhancing recognition ability. Sun et al. [50] introduced the MOBS-TD approach, which attempts to pick bands with improved target separation and robustness, as well as a maximum to submaximum ratio evaluation mechanism that efficiently reduces target false alarms. Furthermore, Fu et al. [51] proposed a structure-preserving and weakly redundant band selection method (SPWR) for hyperspectral imagery, capturing spectral features of heterogeneous regions through hyperspectral imagery segmentation and constructing region-specific multimetric hypergraphs to accurately express the neighboring relationships between bands. Future research will examine enhanced band-selection approaches to improve the accuracy and reliability of farmland non-agriculturalization monitoring.
2. The model’s generalization capacity is critical in various spatial resolutions and environmental settings. Data with varying terrains might result in considerable changes in feature extraction and representation capabilities. Hence, the model must be adaptable to multiple terrains. Given Kaifeng City’s unusual geographic location, models trained on datasets from this region may perform poorly when applied to other terrains like hills or mountains. As a result, future research should include samples from many geographic regions to improve our understanding of complicated non-agriculturalization behaviors.
3. Traditional dataset-construction methods in agricultural change studies typically require many labeled samples, which is especially difficult in resource-constrained environments. When samples from non-farming areas are underrepresented, the model’s performance suffers dramatically, lowering the accuracy of cropland CD. As a result, it is especially crucial to examine the production of pseudo-labels for unlabeled data or to optimize the model via self-learning.
4. Existing CD approaches are primarily concerned with the presence of change, with insufficient attention paid to the type of change and its consequences. The identification of non-agricultural areas not only supports the identification of change areas, but also aids in the definition of the conversion of agricultural land to non-agricultural usage. Semantic information recognition networks can provide a better understanding of the dynamics of non-agricultural areas, resulting in more accurate detection. For example, in urban planning, the model can detect the conversion of agricultural property to commercial or residential land, providing statistical support for planning decisions. In disaster management, early awareness of land use change might aid in developing successful emergency response strategies. As a result, investigating the model’s application potential in these areas will make research more relevant to real applications.

## Figures and Tables

**Table 1:** Information about different datasets.

Dataset	Image Source	Label Category
PX-CLCD	GF2	Buildings, Forest, Roads
SET-CLCD	GF1, GF2	Buildings, Unconstructed Areas
HN-CLCD	ZY3, JL1, GF1, GF2, BJ-2	Buildings, Greenhouses, Lakes, Roads

**Table 2:** Experimental results of different methods on HN-CLCD dataset. Best values are shown in bold. PR represents the Precision; RC represents the Recall; F1 is the composite metric; and IoU represents the Intersection over Union.

Network	PR (%)	RC (%)	F1 (%)	IoU (%)
FC-EF	72.66	58.35	64.73	47.85
SNUNet	72.92	62.39	67.25	50.65
STANet	75.59	59.22	66.41	49.72
LightCD	78.43	62.69	69.68	53.47
HCGMNet	79.94	69.24	74.20	58.99
CGNet-CD	79.63	72.94	76.14	61.48
DDAM-Net	**80.70**	**77.90**	**79.27**	**65.66**

**Table 3:** Experimental results of different methods on PX-CLCD dataset. Best values are shown in bold. PR represents the Precision; RC represents the Recall; F1 is the composite metric; and IoU represents the Intersection over Union.

Network	PR (%)	RC (%)	F1 (%)	IoU (%)
FC-EF	68.15	60.37	64.03	47.09
SNUNet	76.48	78.01	77.24	62.92
STANet	69.98	63.51	66.59	50.91
LightCD	83.0	85.4	84.2	72.6
HCGMNet	89.27	92.87	91.03	83.54
CGNet-CD	93.83	94.70	94.26	89.14
DDAM-Net	**95.47**	**94.78**	**95.12**	**90.70**

**Table 4:** Experimental results of different methods on SET-CLCD dataset. Best values are shown in bold. PR represents the Precision; RC represents the Recall; F1 is the composite metric; and IoU represents the Intersection over Union.

Network	PR (%)	RC (%)	F1 (%)	IoU (%)
FC-EF	63.27	54.06	58.30	41.14
SNUNet	59.53	68.08	63.52	46.54
STANet	65.52	60.48	62.90	45.88
LightCD	71.09	66.78	68.86	52.51
HCGMNet	75.02	**67.89**	71.25	55.34
CGNet-CD	74.52	66.90	70.50	54.45
DDAM-Net	**77.59**	**67.87**	**72.40**	**56.74**

**Table 5:** Relevant settings of ablation experiments on the HN-CLCD. Best values are shown in bold. PR represents the Precision; RC represents the Recall; F1 is the composite metric; and IoU represents the Intersection over Union.

Method	Ablation Designs							
	ResNet	DEFM	CAM	ARM	PR (%)	RC (%)	F1 (%)	IoU (%)
Model-a	**√**	**√**			75.21	69.12	72.03	56.29
Model-b	**√**	**√**		**√**	77.67	75.13	76.38	61.78
Model-c	**√**	**√**	**√**		73.57	76.32	74.92	59.90
Model-d	**√**		**√**	**√**	74.43	72.52	73.46	58.06
Model-e	**√**	**√**	**√**	**√**	**80.70**	**77.90**	**79.27**	**65.66**

## Data Availability

The PX-CLCD and SET-CLCD datasets are available online. The HN-CLCD datasets are available from the corresponding author upon request.
